# Lupus enteritis

**DOI:** 10.11604/pamj.2019.33.205.12325

**Published:** 2019-07-15

**Authors:** Senthil Kumar Aiyappan, Akilesh Suvindran

**Affiliations:** 1 SRM Medical College Hospital and Research Centre, Kattangulathur, Kancheepuram-603203, Tamilnadu, India

**Keywords:** Lupus, enteritis, vasculitis

## Image in medicine

Lupus enteritis is a rare and poorly understood cause of abdominal pain in patients with systemic lupus erythematosus (SLE). We report a patient with this rare condition who was referred to radiology department for CT abdomen examination. A 40-year-old female patient presented with abdominal pain, distension, fever and diarrhea for the past 1 week. Antinuclear antibody was strongly positive showing speckled pattern. The double stranded DNA was positive. Clinically she gave history of joint pain and on examination oral ulcers and malar rash were present. Hence a definitive diagnosis of systemic lupus erythematosus was considered. CT abdomen was done which showed presence of bowel wall thickening, dilatation of intestinal segments, engorgement of mesenteric vessels, increased attenuation of mesenteric fat, ascites, peritoneal enhancement, retroperitoneal lymphadenopathy and target sign which is due to submucosal edema. In view of the patient's medical history of systemic lupus erythematosis (SLE) and the appearance of the bowel, lupus enteritis from vasculitis was diagnosed. Patient was treated with steroids and the patient subsequently improved. Mesenteric vasculitis is uncommon (2.2-9.7%) and highly lethal among gastrointestinal complications of SLE, if it is not carefully diagnosed and promptly treated. CT scanning has become the gold standard for diagnosis of lupus enteritis. The most frequent findings described were the bowel, mesenteric changes, ascites and retroperitoneal lymphadenopathy which our patient had. Lupus enteritis is a rare manifestation of the disease, but is important to suggest as the vasculitis requires control of the disease by high doses of steroids.

**Figure 1:**
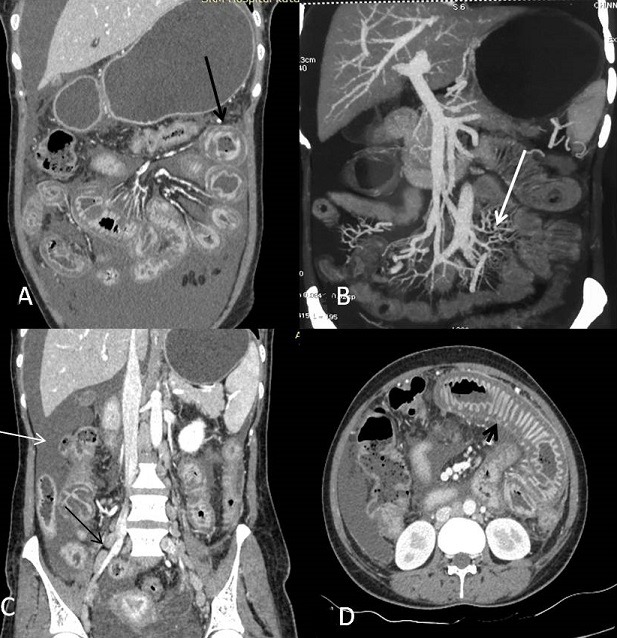
(A) coronal Contrast CT showing target sign (black arrow) with enhancing mucosa and serosa/muscularis propria with diffuse hypoattenuation of submucosa; (B) showing increased vascularity of adjacent mesentery (white arrow); (C) showing retroperitoneal lymphadenopathy (thin black arrow) and ascites (thin white arrow); (D) showing fold thickening of Jejunal loops(short black arrow)

